# Material Monitoring of a Composite Dome Pavilion Made by Robotic Coreless Filament Winding

**DOI:** 10.3390/ma14195509

**Published:** 2021-09-23

**Authors:** Pascal Mindermann, Bas Rongen, Drilon Gubetini, Jan Knippers, Götz T. Gresser

**Affiliations:** 1 Institute for Textile and Fiber Technologies, University of Stuttgart, Pfaffenwaldring 9, 70569 Stuttgart, Germany; goetz.gresser@itft.uni-stuttgart.de; 2 German Institutes of Textile and Fiber Research Denkendorf, Körschtalstraße 26, 73770 Denkendorf, Germany; 3 Institute of Building Structures and Structural Design, University of Stuttgart, Keplerstraße 11, 70174 Stuttgart, Germany; b.rongen.itke@gmail.com (B.R.); d.gubetini@tum.de (D.G.); jan.knippers@itke.uni-stuttgart.de (J.K.)

**Keywords:** robotic coreless filament winding, lightweight dome structure, on-site inspection, thermal imaging, scanning electron microscopy, outdoor weathering, BUGA Fiber Pavilion

## Abstract

A hemispherical research demonstration pavilion was presented to the public from April to October 2019. It was the first large-scale lightweight dome with a supporting roof structure primarily made of carbon- and glass-fiber-reinforced composites, fabricated by robotic coreless filament winding. We conducted monitoring to ascertain the sturdiness of the fiber composite material of the supporting structure over the course of 130 days. This paper presents the methods and results of on-site monitoring as well as laboratory inspections. The thermal behavior of the pavilion was characterized, the color change of the matrix was quantified, and the inner composition of the coreless wound structures was investigated. This validated the structural design and revealed that the surface temperatures of the carbon fibers do not exceed the guideline values of flat, black façades and that UV absorbers need to be improved for such applications.

## 1. Introduction

The use of fiber-reinforced composite materials, such as carbon- and glass-fiber-reinforced plastics (C/GFRPs), in construction has grown in popularity recently [1]. They are extensively used in aerospace engineering [2] due to their superior mass-specific mechanical performance [3]. In times of resource scarcity [4], significant population growth [5], and increasing demand for building floor space [6], a material system that exhibits efficiency and sustainability will allow us to keep up with the required productivity of future decades. Among other advantages, fiber-reinforced composites offer superior corrosive resistance. Composite reinforcement bars for concrete construction benefit from this. Additionally, flat textile composite patches are a promising alternative in the repair of existing buildings [7]. Composites are also deployed as façade elements [8], and they allow for a reduction in concrete coverage [9,10] or offer more design freedom [11]. Pultruded fiber composites for load-bearing structures are currently state-of-the-art products [12] that can be used within general design approvals included by manufacturers. Due to their low weight input, composite building materials may also be suitable for stock extension and urban densification. Apart from material aspects and fabrication processes, design methods also have to be reconsidered in order to compete in the increasingly digitalized [13] construction industry.

Currently, the broad utilization of composites for full-scale supporting structures is missing in various industries. Occasionally, there are isolated implementations of these technologies [14,15,16]. In research and academia, innovative concepts of composite usage for load-bearing structures are demonstrated in small scale projects [17,18]. This has resulted in the first large-scale research demonstration, the fiber pavilion at the Federal Garden Show (BUGA) in Heilbronn, Germany, which exclusively utilizes C/GFRP as a supporting structure. Within this case study [19], developed by the University of Stuttgart, C/GFRP is integrated into a non-conventional building system, and it may be utilized in further iterations for roof or ceiling structures, as well as bridges.

The most prominent advantage resulting from this is a low weight per built floor area that is roughly estimated to be five times lighter than conventional steel structures with a similar span. This simplifies the handling, transport, and assembly of such structures. Apart from this, the support structure can be built up modularly and used again. Each component can be tailored to a specific load case [20]. These components can have a visually appealing freeform, resulting from the combination of the fiber path and the winding frame, which allows architects to fulfill the demand for unique buildings.

**Figure 1:**
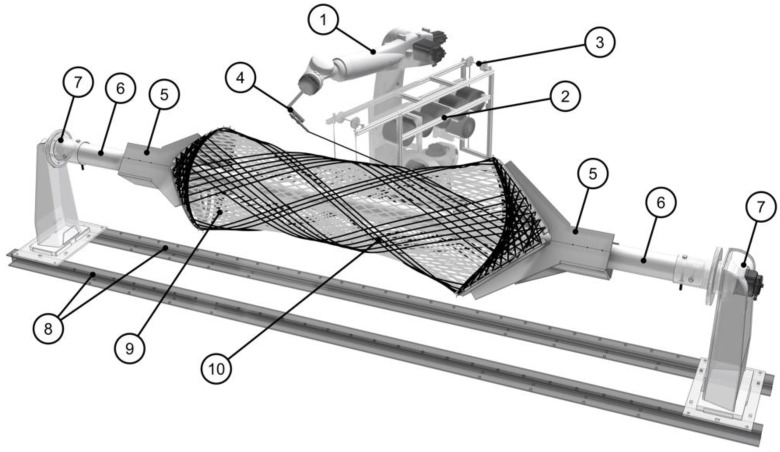
Robotic coreless filament winding setup for the manufacturing of the BUGA composite components: 1, industrial robot (6-axis); 2, robot-mounted creel; 3, fiber tension mechanism; 4, robotic winding end effector; 5, winding frame; 6, continuous steel tube; 7, one-axis positioner (external axis); 8, H-shaped steel beams; 9, underlaying glass fiber body; 10, carbon fiber reinforcement. © ICD/ITKE University of Stuttgart.

The manufacturing process that enables this approach is robotic coreless filament winding (RCFW). This textile manufacturing process evolved from the fabrication of cylindrical-wound composite tanks. Instead of using a mandrel or mold on which to place the fibers, a continuous strand of impregnated fibers is wound around multiple, spatially arranged winding pins by an industrial robot (Figure 1). The fibers span freely between those anchor points, interacting with just each other. This allows for the tuning of the fiber net and thus the structural characteristics of each individual component, without the cost of changes to any hardware. The used thermoset resin needs to be cured in an oven to create the self-supporting composite component. By unscrewing it, it can be taken off the winding frame, which holds the pins in place during production. The only part of the pin that remains in the component is a metallic sleeve, which serves as a load transmission element.

Despite these technological advantages [21], the industrial application of RCFW is not immediately possible in the building sector, as regulatory obstacles still have to be overcome in each individual case. These requirements and approvals in individual cases require an approval process, which is currently accompanied by full-scale testing [22] due to the limited ability to reliably simulate such structures numerically, since the fabrication methodology is new. In Germany, such an approval process can differ between federal states. As part of this, several reports by experts in the field of load-bearing composites were requested. Reports were carried out examining affirmative experimental results with regard to load-bearing capacity, design concept, and fire behavior of the material.

In addition to the mechanical performance of this material system applied in fabricating CFW components, the behavior of the building in its environment over its complete life span is also examined. The objective of this research was to evaluate these points in relation to the BUGA Fiber Pavilion, to ensure that no structurally relevant material changes occurred, and to characterize the material changes that did happen. The findings of this study could therefore assist in shaping future regulations.

## 2. Materials and Methods

### 2.1. The Object of this Study—BUGA Fiber Pavilion

Within the context of this project [23], all phases could be examined successfully, from conceptualization, detailing, and execution to building monitoring. The exhibition ran from 14 April 2019 to 6 October 2019, so the pavilion was planned to be a temporary structure. The site location [24] was at 49°08′48.5″ N, 9°12′22.7″ E, and 157 m above sea level. Heilbronn has a moderate continental climate with mild winters and warm to hot summers. On average, the annual temperature is 10.3 °C and rainfall is 656 mm per year.

**Figure 2:**
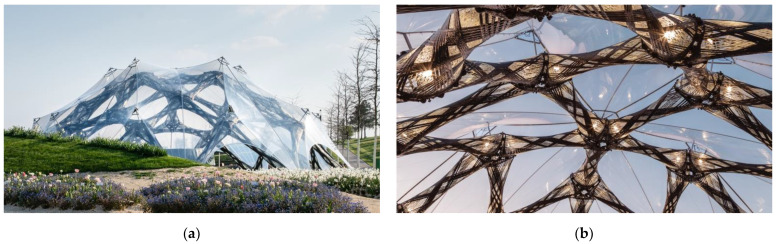
BUGA Fiber Pavilion: (**a**) photograph of the north side exterior of the pavilion during day; (**b**) interior view during twilight. © ICD/ITKE University of Stuttgart.

The pavilion (Figure 2) was designed by two institutes of the University of Stuttgart, the Institute for Computational Design and Construction (ICD) and the Institute of Building Structures and Structural Design (ITKE). The serial production took place at FibR GmbH. The aim of the demonstration was to exhibit how cyber–physical design and fabrication processes can make full use of a novel composite building system to optimize a structure’s material efficiency [25]. Moreover, it was the first time that a building authority authorization process was passed; the “approval in the individual case” was achieved within one year.

The supporting structure of the pavilion consisted of 60 composite components made of C/GFRP by RCFW. Those components were bolted together via winding sleeves to steel angle-shaped connectors. In addition, the connectors served as tolerance compensation [20]. The dome rested on 11 circular-arranged foundations. Above the composite structure, an ethylene tetrafluoroethylene (ETFE) foil was attached to a metallic hinged support, which partially protected the components and 400 m^2^ of the covered surface from wind, sun exposure, and precipitation. The pavilion had a base diameter of 23 m and a maximum height of 7 m, and was distinguished by its low weight per built floor area of 7.6 kg/m^2^.

A key element of the novel BUGA building system was the coreless wound composite component. This was a further development on the previously used Elytra building system [15]. Its advantages are an improved decoupling of the winding frame from the composite component and an increased adaptability of the frame. Thus, the components could be adjusted between fabrication iterations in length and the angles of the flanges could be set. This resulted in six different configurations of the building system used in the BUGA Pavilion. Each component of this building system had a hyperboloid shape. The fiber net formed a tubular shell-like composite structure, with an elliptic cross-section that could be adjusted by the fiber syntax [26]. A component was tested to endure up to approximately 250 kN in axial compression, and it consisted of 1000 m carbon [27] and 1600 m glass [28] fiber paths. Six rovings were placed simultaneously within six hours of winding, followed by a custom curing sequence: five hours of curing at 110 °C, and temperature ramps of 0.8 K/min. The curing sequence started and ended at room temperature. The material system [29] of the component is presented in Table 1. The utilized materials fulfill their technical requirements, which are defined by their specific role in the material system.

The resin mixture [30] is a result of a trade-off between the high glass transition temperature (T_g_) requirements of LH 287 and the lower price of LH 137/138. Bel-91 [31] is a low-viscosity ultraviolet (UV) absorber for synthetic resin systems. The glass fiber bundles were placed first. After completion of the underlaying glass fiber body the carbon fiber reinforcement is placed.

Since this material system cannot be evaluated within the framework of currently existing technical regulations, a project-specific construction permit by the local building authorization was required. The reasons for this were that CFW structures are not consolidated, as in other composite manufacturing processes, but exhibit an additive manufacturing character. In addition, there is no code defining the temperature loads for CFW structures. To cover all safety-relevant aspects regarding the composite materials, an expert report requested a validation of our structural simulations. Several destructive mechanical tests at full-scale were carried out on individual components [22]. To check the joints, an assembly was also tested. The report defined the safety factor for the fiber composite and demanded on-site inspections of the pavilion.

### 2.2. Inspection Methods

Site inspections were carried out on a monthly basis. The entire support structure was inspected visually by a qualified team of two to three persons to establish changes. Only material changes on the surface were observable. In order to document changes, photographs of relevant sections were always taken from the same positions to be comparable.

To follow the yellowing of the matrix system, photographs of the glass fiber bundles were taken with black, grey, and white reference cards. After adjusting the pictures using the cards, the color information for each pixel representing the glass fiber bundles was extracted and translated into LAB color space [32] (L = 0 to 100 = black to white, A = −128 to +127 = green to red, and B = −128 to +127 = blue to yellow).

To record the local weather, relative humidity and ambient temperatures inside and outside the pavilion were recorded at the same distance (approx. 1.7 m) from the ground by the same mobile weather station during site inspections. The weather station was protected from direct sun exposure.

Using a pyrometer, the local temperatures were measured on the interior and exterior carbon and glass fiber bundles, respectively, on the sides facing to and away from the sun. To obtain a spatial overview of temperature distribution, a thermal imaging camera [33] was used to take pictures.

In the design of fiber-reinforced composites, the fiber volume ratio (FVR) is relevant, as it defines structural properties by the rule of mixtures. In particular, in RCFW, the effective fiber cross-section must be considered, as well as the material bundle cross-section. For the purpose of structural simulations, material parameters were calibrated by mechanical full-scale tests with multiple loading scenarios. Later, the FVR of the carbon fiber bundles was investigated by thermogravimetric analysis under a nitrogen atmosphere with a correction factor to compensate for the remaining resin. Deviating from this DIN [34], the fiber mass ratio was calculated directly, as the density of the composite could not be measured experimentally.

Impregnation quality was investigated by scanning electron microscopy (SEM) scans on specimens that were cut perpendicular to the fiber orientation on an abrasive cutting saw, and they were polished after being cast in resin. The specimens were extracted from the retaining samples that were wound simultaneously with the component, and they were later cut off.

### 2.3. Theoretical Expectations

The determination of the partial safety factors for our fiber composites is based on the recommendation of BÜV (Bau-Überwachungsverein e.V.) [35]. One parameter was the negative impact of an increased temperature [36] on the performance of the matrix and, therefore, the whole structure. Even minor effects could lead to structurally relevant issues. It was expected that the temperatures of the carbon fiber bundles would be significantly higher than those of the glass fibers. This is due to the different absorptivity of the two materials (glass = 0.24, carbon = 0.91) [37]. The temperature of the carbon fibers should not exceed 79 °C for a black façade [38]. It was expected that the temperatures inside the dome would be only slightly higher, since the foil should not have a greenhouse effect and should not significantly impede air flow. The water fountains near the pavilion were not expected to have any effect on the structure. Yellowing was expected to be insignificant due to the UV absorber that was added to the resin mixture, as per the manufacturer’s specification. The FVR was expected to be around 50%, since this was characteristic for RCFW in previous structures. The impregnation was expected to be good and the number of cavities was expected to be negligible.

## 3. Results

### 3.1. On-Site Inspections and Building Monitoring

During the pavilion’s lifespan, five site inspections were conducted. No structurally relevant material changes could be found by visual inspections on the composite components, the foil, the foundations, or the assembly interfaces. This included the delamination of fibers, the buckling of reinforcement strands, cracking of the resin, uncovered fibers, significant global deformations, contamination, manipulation, or attachments of any kind. An influence of the water spray from the nearby fountains could not be detected.

No changes in the supporting structure were noticed. Precise examinations by means of a total station or photogrammetry were deemed not of interest as the pavilion is a lightweight structure with a limited life span, in which long term effects such as creep and settlements play a limited role.

#### 3.1.1. In Situ Measurements

**Figure 3:**
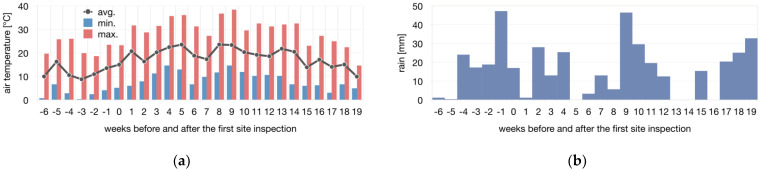
Dataset of the external weather station in weeks before and after the first site inspection: (**a**) average, minimum, and maximum temperatures per week; (**b**) accumulated rainfall per week. Data obtained from [39].

A dataset [39] from an official permanent weather station in Heilbronn was obtained (Figure 3). During the BUGA, the average air temperature was 1.1 K higher and rainfall increased by 41.7 mm compared to average values [39]. The pavilion experienced 420 mm of rain, 1303 h of sunshine, an average windspeed of 9.1 ± 3.5 km/h, and a maximum wind speed of 22 km/h, with winds coming primarily from the south (171.5 ± 67.6°) [39].

**Figure 4:**
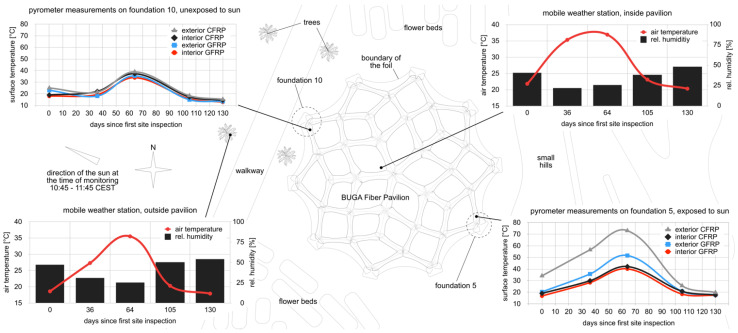
Floor plan of the BUGA Fiber Pavilion. The in situ measurements are indicated at their locations.

Over the course of 130 days, starting on 23 May 2019, in situ measurements with pyrometers and a mobile weather station were conducted (Figure 4). The ambient temperature reached a ceiling of 36.9 °C, and the lowest relative humidity was 22%. The air temperature and humidity patterns (Figure 3 and Figure 4) can be explained by seasonal variations. On average, the air inside the pavilion was measured to be 15.5 ± 9.1% hotter and 14.6 ± 12.8% drier. This is a greenhouse effect caused by the ETFE foil.

The surface temperature of the lower composite components was measured with a pyrometer, with reference points on the inner and outer sides of the component and on opposite sides of the pavilion (Figure 4). The temperature for glass fiber bundles ranged from 12.9 °C to 51.6 °C and from 14.9 °C to 73.0 °C for the carbon fiber bundles. For the components exposed to direct sunlight, surface temperatures between inside and outside differed by a factor of four; without direct sunlight, the temperatures were similar. The more intense the solar radiation, the greater the differences. The external carbon fiber bundles responded the strongest to solar radiation, followed by the external glass fiber bundles.

In direct sunlight, the glass is only 17.4 ± 19.8% warmer than its surroundings. Carbon is significantly more sensitive to solar radiation, with an increase of 66.5 ± 45.3%. This is a result of different absorption coefficients. The high deviation in carbon can be explained by the small difference during the colder inspection days based on the seasons. Although a color change could only be observed in the glass fiber matrix, this means that the thermal loads in the epoxy matrix are correspondingly higher for carbon than they are for glass fiber bundles. The deterioration of the resin due to UV exposure should be independent of the type of fiber.

The maximum temperature difference between carbon and glass fiber bundles within one component is 33.1 K, due to the different thermal expansion components. Due to the extending glass fibers, the carbon fibers received tensile stress. If heated sufficiently, exposed glass fibers would buckle outward. The glass fiber formwork is enclosed by carbon fiber reinforcements. This arrangement prevents delamination between the layers. Effects due to thermal expansion could not be observed.

On average, the carbon was 22.9 ± 8.6 °C in shadow and 41.8 ± 22.3 °C in sunshine. For glass, it was 20.5 ± 8.0 °C and 29.2 ± 14.4 °C in solar exposure. On the unexposed side of the component facing the sun, the temperature of the carbon fibers was 3.0 ± 4.0 K; this was 3.7 ± 4.8 K higher in relation to glass fiber bundles. This is the result of heat conduction within the component.

#### 3.1.2. Thermal Distribution Monitoring

**Figure 5:**
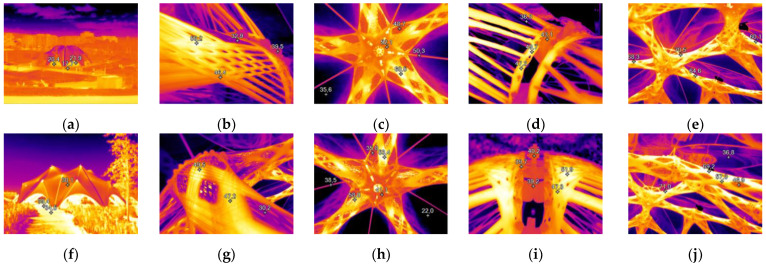
Selection of representative infrared pictures of the BUGA Fiber Pavilion. Temperature markers indicate °C: (**a**,**f**) view of the pavilion as a whole, showing higher ground temperature below the pavilion (scale 4.9–24.8 °C, 4.9–56.3 °C); (**b**,**g**) detailed view of a composite component to show difference between carbon and glass (scale 18.4–58.9 °C, 17.0–56.1 °C); (**c**,**h**) center element of the pavilion with thermal reflection in the foil (scale 35.7–64.7 °C, 23.9–59.4 °C); (**d**,**i**) metallic load transmission elements not thermally interfering with the composite (scale 33.4–52.3 °C, 19.4–51.6 °C); (**e**,**j**) view of the upper components with maximum measured temperature (scale 10.6–71.3 °C, 9.1–71.6 °C).

The distribution of the surface temperature could be captured for several vantage points (Figure 5). The ground temperature below the pavilion was measured to be 19% higher than in the surrounding area. Carbon fiber bundles have a 47% higher temperature than adjacent glass fiber bundles. The ETFE foil reflected thermal radiation, which can be seen on the inside of the pavilion. The foil connection elements are 58% hotter than the foil itself. It could be observed that there was a vertical temperature gradient due to a heat trap effect engendered by the foil.

**Figure 6:**
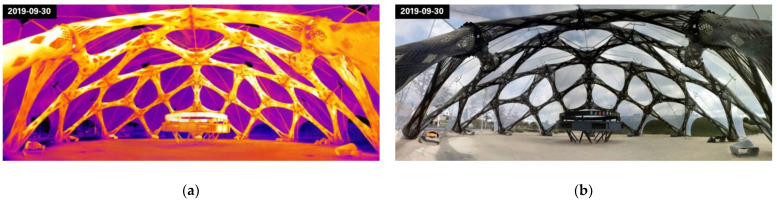
Stitched panoramic pictures of the BUGA Fiber Pavilion. Sun position towards the right side of the picture: (**a**) infrared spectrum (scale 10.0–23.0 °C); (**b**) visual spectrum.

The metallic connections did not disturb the temperature distribution around the load transmission elements. The metal elements were 17% colder than the carbon fibers and 19% hotter than the glass fibers. The temperature distribution over the pavilion depended primarily on sun exposure (Figure 6). Areas that were not covered by the ETFE foil were exposed to direct sunlight and reached higher temperatures.

The highest temperature captured by the pyrometer during site inspections was 73.0 °C (Figure 4). It was measured at the exposed carbon reinforcement of foundation five on 26 July 2019. The outside air temperature was 35.5 °C and it was cloudless. By taking several pictures with the thermal camera and stitching them into a single frame, a vertical rise in temperature of 2.5 ± 2.0 K was found for the carbon fiber reinforcement.

Based on the highest temperature captured by the pyrometer, the highest temperature appearing in the uppermost components could be estimated to be 73.0 °C + 2.5 K = 75.5 °C. This is slightly higher than the 71.0 ± 2.0 °C measured by the thermal camera for this location directly.

#### 3.1.3. Yellowing of the Thermoset Matrix System

**Figure 7:**
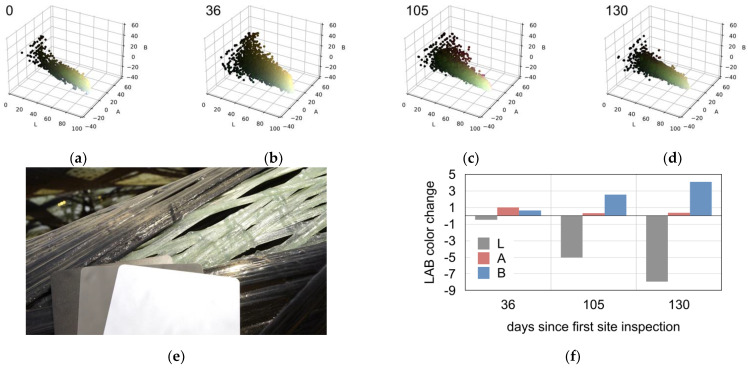
Monitoring results of the composite yellowing: (**a**–**d**) plots of the pixel colors in LAB color space for inspection, days 0, 36, 105, and 130. (**e**) Example picture with reference cards. (**f**) Relative changes in averages for LAB values (L = 0 to 100 = black to white, A = −128 to +127 = green to red, B = −128 to +127 = blue to yellow).

The yellowing of the matrix could be easily recognized on the glass fiber bundles towards the end of the inspection period. This change is caused by degradation of the resin system due to environmental conditions, predominantly UV irradiation. The ETFE foil offers little protection against UV light, and is more effective against rain. The UV also ages the matrix of the carbon fiber bundles; here, the yellowing was not visible since carbon is black. The average LAB color values were calculated for each site inspection appointment, revealing that there was a significant change in the B (blue to yellow) value from −8.82 to −4.75, which represented a yellowing (Figure 7). The lightness value slightly decreased from 81.85 to 73.90, while the A (green to red) value stayed nearly the same (from −7.73 to −7.35) over the course of all the site inspections.

### 3.2. Material Inspection on the Supporting Structure

As a measure of quality control for the industrial fabrication of the pavilion, material samples were wound simultaneously to the fabrication of the full-scale building component and kept as retaining samples. Those fiber loops were investigated during the preparation of the expert report. Inspections of the bundle cross-sections were performed primarily on the carbon fiber bundles, as they were the supporting structure of the composite elements.

#### 3.2.1. Fiber Volume Ratio Determination

On average, the FVR measured by pyrolysis on the BUGA C5_01 [26] retaining sample for the carbon fiber bundles was 36.7 ± 1.7% (seven samples); this was 50.0 ± 1.3% (six samples) for glass fiber bundles. By weighing a component identical to a BUGA C5 component and by calculating the total fiber amount, by summing up all lengths of each syntax, the FVR of such a component can be determined after manufacturing. The measured component with a total mass of 65.86 kg contains 22.36 kg of carbon and 14.72 kg of glass fibers based on their digital fiber path length and linear densities. The mass of the sleeves came to 2.4 kg, and the total resin mass could be calculated as the remainder, resulting in an FVR of 47.5% for the whole component. In comparison to the average value of 44.7% for CFRP and GFRP obtained by pyrolysis, this demonstrates that both values match. The deviation in FVR between different specific locations demonstrates that there is inhomogeneous resin distribution along the fiber path. This is a result of the winding process and the syntax. Crossing points, hooking points, and free-spanning segments have varying FVRs due to differences in tension and local fiber geometry.

#### 3.2.2. SEM Scans on Fiber Bundle Cross-Sections

**Figure 8:**
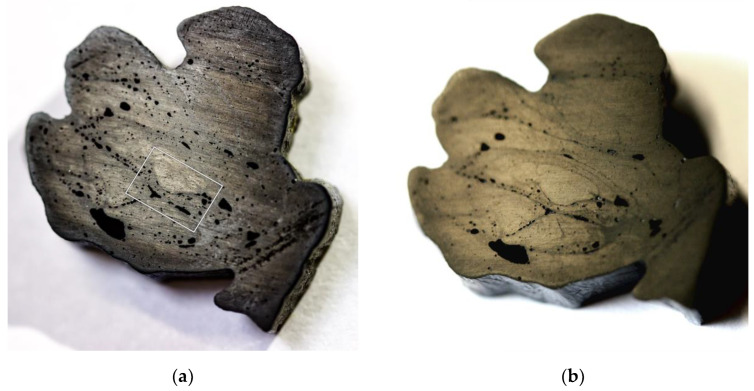
Cross-sectional view of a BUGA carbon-fiber-bundle-retaining sample containing 48 × 6 48K rovings: (**a**) picture with increased contrast and sharpness to make voids easily visible. Box shows SEM scan area of Figure 9. (**b**) Picture with oblique yellow lighting revealing resin bridges (matte) and rovings (shiny).

The retaining samples of the carbon fiber bundles were cut and photographed (Figure 8), as well as examined by SEM. The impregnation quality within the roving bundles was excellent among all samples. Fiber orientation was found to be perpendicular to the section’s orientation, which can be seen as the filament cross-section being circular and not elliptical [40].

**Figure 9:**
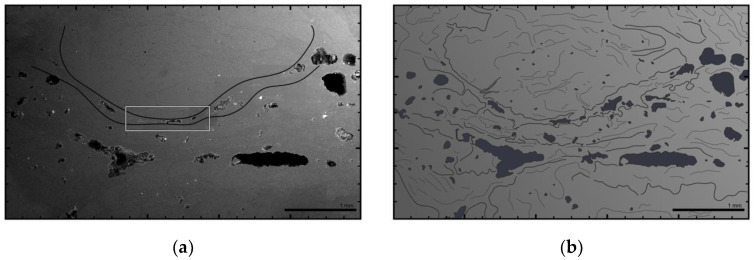

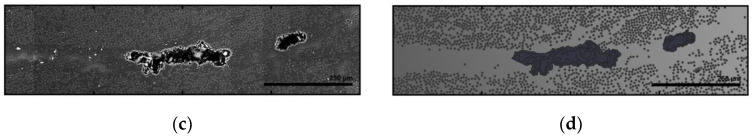
SEM scan of the fiber bundle interface area cross-section: (**a**) SEM scan from Figure 8 with indication of the interfaces between rovings; (**b**) schematic analysis of the same area, with voids and inner roving structures marked; (**c**) enlargement of the framed area in subfigure (**a**); (**d**) schematic analysis of subfigure (**a**) with marked voids and single carbon fiber filaments.

Consolidation quality fluctuates within a certain bandwidth, and it is recognizable by the size, number, and distribution of defects (Figure 9). Within a roving bundle, consolidation quality is higher due to the applied compression by passing it through the winding end effector. Between successively placed layers of fiber bundles, consolidation is worse.

The fiber bundles carry additional amounts of resin with them on the outer surface. If compression applied by the fiber–fiber interaction during the CFW process is not sufficient, the excess resin ends up between the fiber bundle layers and forms an interface. These are characterized by pure-resin layers and lined-up air-filled cavities, both of which are visible in cross-sections, even by the naked eye (Figure 8). This underlines the additive manufacturing character of RCFW.

On the outer surface of the fiber bundles, a resin layer is also present, which protects the fiber from environmental impacts. Here, remaining broken filaments can also be recognized due to their deviating orientation. The number of the voids negatively correlates with their size for all measured samples, similar to [41]. The porosity measured on the samples is 3.14 ± 1.98% of the cross-section area.

## 4. Discussion

In the future, a fibrous temperature sensor should be implemented in the relevant locations of the building, and it should cover exposed carbon fiber bundles and unexposed glass fiber bundles within the same component. This would allow for the continuous monitoring of the thermal behavior of the composite dome. Such a sensor should be implemented in a way that interferes neither structurally nor visually. A permanent on-site weather station should be integrated to record climate data. These measures would allow for more continuous monitoring and the capturing of short time events, and would thus allow for a deeper understanding of the building’s thermal behavior.

Any contactless thermometry is dependent on the emissivity and background temperature settings of devices. In the case of the used thermal imaging camera, these para-meters could be adjusted in post-processing. Background temperatures were taken from the mobile weather station measurement of the corresponding day. In the case of the pyro-meter, the emissivity coefficient was fixed at 0.95. The temperatures between the components measured with the pyrometer in the shade of the pavilion were 15% lower than the temperatures measured by the mobile weather station.

The FVR for the carbon fiber syntax was shown to be unexpectedly low. The number of voids was higher than expected but without structural relevance in this project, since they were already included in the structural simulations that were calibrated by full-scale mechanical destructive tests on similar components. The same applies to the pure-resin layers. With the currently used design methods, the fibers are not sufficiently compressed. In this case, excess resin is useful as a means by which to fill cavities and distribute the force flow more homogeneously by connecting adjacent bundles. The methods used in other textile processes, such as the use of a vacuum to suck out excess resin or press it out in a mold, are difficult to transfer to CFW due to the lattice component structure.

## 5. Conclusions

Based on the visual inspections related to the monitoring, the validity of the structural design can be confirmed. Including mechanical testing, this study validated the safety factor for the design concept, as specified in the expert report. The monitoring concept could also be effective and necessary for future projects. This verification will help to include RCFW buildings in official regulations in the future, which is beneficial from a material point of view.

The observed yellowing of the resin revealed that its environmental protection aspects need to be improved for outdoor projects involving aesthetical use of GFRP. Since the heat flux between carbon and glass fibers is not sufficient to homogenize the temperature difference during daytime, possible mechanical stresses caused by differences in thermal expansion need to be considered in future projects with a hybrid fiber system, especially in warmer contexts. Linked simulations on sun exposure could reveal bending stresses.

The maximum temperature measured on the carbon fiber reinforcement corresponds with the theoretical value of 79 °C given by the regulations for flat black façades [38]. This regulation could be extended to C/GFRP building systems made by RCFW. This is crucial for future resin system selections. The T_g_ of the resin used was 106 °C, which was significantly higher than the maximum temperature observed at which strength decreases even before reaching the T_g_ of the resin. The impact of covering foils on the inside air temperature of such buildings also requires further investigation.

As long as impregnation and fiber orientation are sufficient, any defects that vary the density or distribution of the filaments or fiber bundles from the idealized model are without structural relevance for mesoscopic mechanical design, since the performance loss per cross-section area has to be inherent for all material parameters used. Therefore, a full-scale mechanical test of the component is needed to calibrate the simulation.

In this project, it was possible for the first time to gather monitoring data on a large-scale RCFW building demonstration, which was exposed to a central European climate over 5 months and 20 days. The methodological groundwork for the systematic material monitoring of RCFW established in this project will help to widen the sphere of RCFW and the structural relevance of C/GFRP materials in the engineering field, especially in relation to future certified construction applications. An accurate dataset describing the fabrication process has proven itself to be an invaluable tool for more comprehensive process analysis and monitoring.

## Figures and Tables

**Table 1:** Overview of the material system used for the fabrication of the BUGA component.

Used Raw Material	Processing Method	Internal Interaction	Intended Function	Environmental Interaction	Form-Defining Characteristics
Teijin Tenax-E STS40 F13 48K 3200tex	Robotic coreless filament winding and thermal curing	Mutual displacement due to tension and friction, becoming a composite	Reinforcement of the component	Insignificant	Lattice fiber composite structure conforming to the BUGA building system
Owens Corning PipeStrand S2300 2400tex LS BP11 S CF A			Shaping of the fiber body by pushing carbon fiber outwards		
71.9 wt % Hexion Epikote MGS LR 135, 16.4 wt% Hexion Epikure MGS LH 137/138, 8.8 wt % Hexion Epikure MGS LH 287			Matrix of the composite, adhesive joint to the sleeves	Aging due to sun exposure, etc.	
2.9 wt% HP-Textiles BEL-91			Yellowing inhibitor		
Aluminum, EN AW 6082	Sleeves mounted by bolt connection	Adhesive joint with C/GFRP	Winding pins and force transmission sleeves	Insignificant	
Steel, S355MC (1.0976)	Angles mounted by bolt connection	Frictional connection with the sleeves	Connector and tolerance compensation		Angles cold-formed from planar material

## Data Availability

Publicly available datasets were analyzed in this study. This data can be found here: https://meteostat.net/de/place/DE-OSL9, accessed on 28 August 2021.
